# Obese Individuals With and Without Phlegm-Dampness Constitution Show Different Gut Microbial Composition Associated With Risk of Metabolic Disorders

**DOI:** 10.3389/fcimb.2022.859708

**Published:** 2022-06-01

**Authors:** Juho Shin, Tianxing Li, Linghui Zhu, Qi Wang, Xue Liang, Yanan Li, Xin Wang, Shipeng Zhao, Lingru Li, Yingshuai Li

**Affiliations:** ^1^School of Traditional Chinese Medicine, Beijing University of Chinese Medicine, Beijing, China; ^2^Institute of Basic Theory for Chinese Medicine, China Academy of Chinese Medical Sciences, Beijing, China; ^3^National Institute of Traditional Chinese Medicine Constitution and Preventive Treatment of Diseases, Beijing University of Chinese Medicine, Beijing, China; ^4^People’s Medical Publishing House Co., Ltd., Chinese Medicine Center, Beijing, China; ^5^Sanbo Brain Hospital of Capital Medical University, Beijing, China

**Keywords:** phlegm-dampness constitution, gut microbiota, obesity, obesity subtypes, 16S rRNA

## Abstract

**Background:**

Obesity is conventionally considered a risk factor for multiple metabolic diseases, such as dyslipidemia, type 2 diabetes, hypertension, and cardiovascular disease (CVD). However, not every obese patient will progress to metabolic disease. Phlegm-dampness constitution (PDC), one of the nine TCM constitutions, is considered a high-risk factor for obesity and its complications. Alterations in the gut microbiota have been shown to drive the development and progression of obesity and metabolic disease, however, key microbial changes in obese patients with PDC have a higher risk for metabolic disorders remain elusive.

**Methods:**

We carried out fecal 16S rRNA gene sequencing in the present study, including 30 obese subjects with PDC (PDC), 30 individuals without PDC (non-PDC), and 30 healthy controls with balanced constitution (BC). Metagenomic functional prediction of bacterial taxa was achieved using PICRUSt.

**Results:**

Obese individuals with PDC had higher BMI, waist circumference, hip circumference, and altered composition of their gut microbiota compared to non-PDC obese individuals. At the phylum level, the gut microbiota was characterized by increased abundance of *Bacteroidetes* and decreased levels of *Firmicutes* and *Firmicutes/Bacteroidetes* ratio. At the genus level, *Faecalibacterium*, producing short-chain fatty acid, achieving anti-inflammatory effects and strengthening intestinal barrier functions, was depleted in the PDC group, instead, *Prevotella* was enriched. Most PDC-associated bacteria had a stronger correlation with clinical indicators of metabolic disorders rather than more severe obesity. The PICRUSt analysis demonstrated 70 significantly different microbiome community functions between the two groups, which were mainly involved in carbohydrate and amino acid metabolism, such as promoting Arachidonic acid metabolism, mineral absorption, and Lipopolysaccharide biosynthesis, reducing Arginine and proline metabolism, flavone and flavonol biosynthesis, Glycolysis/Gluconeogenesis, and primary bile acid biosynthesis. Furthermore, a disease classifier based on microbiota was constructed to accurately discriminate PDC individuals from all obese people.

**Conclusion:**

Our study shows that obese individuals with PDC can be distinguished from non-PDC obese individuals based on gut microbial characteristics. The composition of the gut microbiome altered in obese with PDC may be responsible for their high risk of metabolic diseases.

## Introduction

Over the past 40 years, obesity has become a substantial health economics issue because of its high-risk factor of dyslipidemia, type 2 diabetes, hypertension, and cardiovascular disease (CVD) (2016; Grieve et al., 2013; O'Neill and O'Driscoll, 2015). However, not all obese individuals exhibit characteristics of metabolic disorders. Some obese people are highly sensitive to insulin, have normal blood pressure, average blood glucose, and normal lipid levels. They are often referred to as metabolically healthy obesity (MHO) (Hinnouho et al., 2013; Stefan et al., 2013). A meta-analysis of data from 12 cohorts and seven intervention studies found that almost one-third of obese individuals were metabolically healthy (Lin et al., 2017). Therefore, accurate identification of metabolic abnormalities in obese patients, or those at risk for metabolic abnormalities, and individualized prevention are essential. Over the years, in many independent clinical studies, investigators have typed obesity. One category is based on obesity phenotypes such as BMI, waist circumference, waist-to-hip ratio, and visceral fat content; the other category is based on concomitant metabolic markers such as blood pressure, fasting glucose, triglycerides, (TG), and high-density lipoprotein cholesterol (HDL-C); in addition, some scholars have used homeostasis models to assess insulin resistance or insulin sensitivity (Matthias, 2020). The diversity of obesity typing methods reflects not only the importance of this clinical phenomenon but also the difficulty of typing studies and the need to expand new perspectives in order to get an earlier and more accurate picture of the population at risk for metabolic disorders of obesity.

Traditional Chinese medicine (TCM) is typically individualized medicine, which emphasizes the idea of “tailoring to the individual” and determines the phenotype by summarizing the signs and symptoms exhibited by the patient to guide the choice of treatment. As early as in the *Inner Classic of Yellow Emperor*, the foundation of Chinese medicine, it is recorded that obesity can be divided into “*lipid-fat*”, “*oily-fat*”, and “*muscular-fat*” based on body shape and lipid muscle distribution. Among them, *oily-fat* people are similar to the abdominal obese people described in modern medicine and more susceptible to various metabolic diseases. The discipline of TCM constitution (TCMC), which inherits the TCM idea of “tailoring to the individual”, divides people into nine types, including one BC and eight unbalanced constitutions (qi-deficiency constitution, yang-deficiency constitution, yin-deficiency constitution, phlegm-dampness constitution, dampness-heat constitution, blood stasis constitution, qi stagnation constitution, and inherited special constitution), these different constitutions with different susceptibility to diseases (Wang, 2019). PDC is caused by the dysfunction of water metabolism in the body and the coalescence of phlegm and dampness, which manifests itself as fat and flabby abdomen; sticky feeling in the mouth; phlegm in the chest; sweaty, oily forehead; bloated pouch; and thick tongue coating. Many factors influence the formation of phlegm-damp constitution, including heredity or excessive consumption of greasy and sweet foods, as well as long-term handling of humid environments, etc., resulting in impaired spleen function and imbalance of body fluid metabolism. TCM believes that the spleen plays a significant role in the digestive system that produces Qi and blood from digested foods and governs water transportation in the body, similar to the stomach and the small or large intestine. Several studies have shown that PDC is a high risk factor for metabolic diseases (Wang et al., 2011), as the “Common Soil” for cerebrovascular accidents, coronary heart disease, diabetes, hypertension, metabolic syndrome, polycystic ovary syndrome, and sleep apnea syndrome. An epidemiological study (Zhu et al., 2010) based on the relationship between TCMC and overweight/obesity in 18,805 adults in China showed that the risk of obesity was significantly higher in the PDC population (OR=2.05, 95% CI, 1.79-2.35). Multi-omics studies have found that PDC populations present metabolic disorder-related single-nucleotide polymorphisms (Wang et al.) (SNPs), transcriptomic features, DNA methylation modifications (Yao, 2016), and metabolomic features (Li et al., 2016). Further studies revealed significant differences in transcriptome expression profiles and metabolome profiles between obese populations with or without PDC. Obese individuals with PDC exhibit a more significant molecular profile related to metabolic disorders and higher insulin resistance, inflammatory response, and oxidative stress levels. Thus, identifying PDC may be a viable approach to screening high-risk subgroups in obese communities.

It is generally accepted that the human metabolic phenotype is determined by the human genome inherited from the parents. Still, in recent years there is increasing evidence that human commensal bacteria, especially commensal gut flora, has a regulatory effect on the metabolic phenotype of the host (Guarner and Malagelada, 2003). A lot of evidence supports an essential role for the gut microbiota in the progression of obesity and its complications (Boulangé et al., 2016; Peters et al., 2018). Patients with obesity exhibit marked alterations in the structure and composition of the gut microbiota, but the richness (Cotillard et al., 2013) of the gut microbiota decreases with the severity of metabolic complications (Aron-Wisnewsky et al., 2019). A European MetaCardis cohort study showed that gut bacterium 2 (bact2) enrichment in patients with severe obesity was associated with inflammatory markers (Vieira-Silva et al., 2020). Some researchers found that (Gao et al., 2018) obese patients with acanthosis nigricans (AN) had worse metabolic status and a lower microbiota diversity than patients without AN. The above studies suggest that the gut microbiota plays a vital role in obesity development and is also a sensitive indicator for identifying metabolic disorders complicating obesity.

Based on the above studies, we speculate that obese people with PDC may have different microbial composition and structure from those with non-PDC. This differential gut microbiota may contribute to the occurrence of severe metabolic disorders. Our study analyzed the gut microbial characteristics of obese people with or without PDC through pyrosequencing of the 16S rRNA gene, compared with healthy individuals with the BC. We compared their gut microbiota and predicted the functional potential of the bacterial community to explore the association between gut microbiota in obese populations with PDC/non-PDC and obesity complications. In addition, gut microbial abundance was used to construct a disease classifier to distinguish people with PDC from obese individuals. This study helps reveal the microecological mechanisms of the obesity-related constitutions. It has important implications for developing individualized intervention programs targeting intestinal flora to prevent and treat metabolic disorders effectively.

## Materials and Methods

### Study Design and Fecal Sample Collection

We recruited 30 cases each for the three groups of obese participants with the PDC and non-phlegm-damp constitution (non-PDC), and standard BMI participants with BC through poster posting and social media distribution from Beijing, China. All the subjects in the current work were strictly enrolled and all of them meet the criteria for determining the PDC/non-PDC/BC constitution (ZZYXH/T157-2009) and pass the professional review. Obesity diagnosis was based on the Chinese Diagnostic Criteria for Adult Obesity, with 18.5≤BMI<24 as standard. BMI≥28 indicated obesity. Subjects were excluded if they had gastrointestinal diseases, malignant tumors, autoimmune disorders, infectious diseases, renal dysfunction, a history of weight loss treatment in the previous year or were administered antibiotics, probiotics, gastrointestinal motility drugs in the previous 1 month. Females who were breastfeeding pregnant or preparing for pregnancy were also excluded.

All clinical information was collected according to standard procedures (detailed in **Additional File 1: Supplementary Methods**). Peripheral venous blood was drawn after subjects were enrolled in the study and participants were given a stool sampler and provided detailed illustrated instructions for sample collection. Stool samples freshly collected from each participant were frozen overnight in liquid nitrogen and stored in a -80°C refrigerator for freezing. After all collections were completed, all samples were couriered to Shenzhen Microbiota Technology Co. for high-throughput sequencing of the same batch.

### DNA Extraction and 16S rRNA Sequencing

Follow the instructions of EZNA^®^ soil kit (Omega Bio-tek, Norcross, GA, US) to extract total DNA from stool samples. Use NanoDrop2000 to detect and calculate the DNA purity and concentration, and use 1% agarose gel to determine the quality of DNA extraction. The 338F (5’-ACTCCTACGGGAGGCAGCAG-3’) and 806R (5’-GGACTACHVGGGTWTCTAAT-3’) primers were used for PCR amplification of the V3-V4 hypervariable region of the sample DNA. The amplification system was 20μl, including sterile double distillation Water 9μl, 5*FastPfu buffer 4μl, 2.5mM dNTPs 2ul, 5μM primer each 0.8μl, FastPfu polymerase 0.4μl; DNA template 10ng. The program was set as follows: 95°C pre-denaturation 3min, 27 cycles (95°C denaturation 30s, 55°C annealing 30s, 72°C extension 45s), 72°C extension 10min (PCR instrument: ABI GeneAmp^®^ 9700). The PCR product was recovered using 2% agarose gel, and the recovered product was purified using AxyPrep DNA Gel Extraction kit (Axygen Biosciences, Union City, CA, USA), eluted with Tris-HCl, and detected by 2% agarose electrophoresis. QuantiFluor™-ST (Promega, USA) was used to detect and quantify DNA. According to the standard operating procedures of the Illumina MiSeq platform (Illumina, San Diego, USA), the purified amplified fragments were subjected to PE 2*300 library construction.

### Sequencing Data Analysis

The paired-end reads were generated and assigned to each sample based on their barcodes and then were merged with Flash (version 1.2.11) software (Reyon et al., 2012). High-quality filtering that reads with ambiguous, homologous sequences or below 200bp were abandoned of the raw tags was conducted to acquire clean tags using split_libraries (version 1.8.0) software of Qiime (version 1.8.0) (Caporaso et al., 2010). The downstream bioinformatic analyses were performed with EasyAmplicon v1.0 (Liu et al., 2021). We then discarded low-abundance sequences (n<8) using the –derep_fullength command of VSEARCH (v2.15) (Rognes et al., 2016). The nonredundant sequences were denoised into amplicon sequence variants (ASVs) *via* the -unoise3 command of USEARCH (v10.0) (Edgar, 2010). The feature ASV table was created with Vsearch –usearch_global. We analyzed high-quality reads with USEARCH, removing chimeric and organelle sequences, to produce 12061 ASVs. Classification of representative sequences for each ASV was applied, and then Ribosomal Database Project (RDP) classifier (version 11.5) (Cole et al., 2014) was used to assign taxonomic data to each sequence. The sequences of all samples were rarefied to 16000 for the downstream diversity analysis. α-diversity was assessed using the species richness indexes and species diversity Shannon indexes. Beta diversity calculations were performed by principal coordinate analysis (PCoA), and the Adonis test was applied to test for significant differences between groups. Heat maps were constructed based on the Wilcoxon rank-sum test (p < 0.05, q < 0.05) at the ASV level. Random forests were used to develop classifier and predict PDC and non-PDC from obesity individuals based on the important microbiota. The microbiota markers proportion data served as input data. The classification performance of each bootstrap was calculated and the area under curve (AUC) was calculated and plotted. Random forests and AUC were performed on oebiotech platform (https://cloud.oebiotech.cn/task/), a free online data analysis website. PICRUSt (performed on the ehbio online platform, http://www.ehbio.com/ImageGP/index.php/Home/Index/index.html) was utilized to predict the metagenomic functional compositions.

### Statistical Analyses

GraphPad Prism 9.0 was used for clinical data analysis. Significance was set at α = 0.05, and all tests were two-tailed. Continuous, Gaussian distributed variables among the three groups were evaluated by one-way ANOVA followed by Tukey’s test for multiple comparisons. Non-Gaussian distributed variables or ranked data were evaluated by the Kruskal-Wallis H-test. Categorical variables were compared by the χ2 test. Raw data were analyzed R software (Version 4.0.3), taxon, and KO modules was tested by Wilcoxon rank sum test, and P values were corrected for multiple testing with the Benjamin & Hochberg method. Pathways that were different in abundance between two groups were obtained using Welch’s t-test. STAMP software (v2.1.3) was utilized for statistical analyses and visualization of the identified pathways. The spearman correlation between the relative abundances of the altered taxon and clinical data was performed on oebiotech platform (https://cloud.oebiotech.cn/task/).

## Results

### Summary of Clinical Characteristics

Compared to healthy controls in BC, the obese people in PDC and non-PDC groups showed higher weight, BMI, waist circumference, hip circumference, and disruptions in glucose and lipid metabolism and an increased inflammatory state. Obese subjects with PDC and obese subjects with non-PDC were matched for obesity characteristics, metabolic characteristics, and inflammatory status. The obese patients with PDC had higher BMI, waist circumference, and hip circumference. Despite the obese individuals with PDC being more obese, there was no difference in blood pressure and blood glucose, insulin levels, nor levels of HDL-C, LDL-C, TG, TC or hs-CRP, UA, or FFA between groups. In addition, there were no significant differences in age and sex matching between the three groups (**Table 1**).

**Table 1:** Characteristics of the study participants.

Characteristics	PDC obesity group (*n*=30)	non-PDC obesity group (*n*=30)	BC control group (*n*=30)	*P*-value
Sex				0.875
Male	13	15	14	
Female	17	15	16	
Age				0.515
25-35	10	11	13	
36-45	20	19	17	
Height(cm)	167.20 ± 9.249	167.50 ± 8.484	167 ± 9.186	0.970
Weight (kg)	90.75 ± 13.74	85.18 ± 9.383^*^	60.33 ± 8.08	<0.001
BMI (kg/m^2^)	32.39 ± 3.837^*^^#^	30.38 ± 2.389^*^	21.54 ± 1.592	<0.001
Body fat (%)	34.67 ± 5.249^*^	32.63 ± 5.476^*^	24.35 ± 5.991	<0.001
VFI	16.5(8,27) ^*^	14(9,25) ^*^	5(2,10)	<0.001
SBP	129.1 ± 12.04	124.2 ± 14.1	122.9 ± 14.05	0.176
DBP	86.73 ± 9.896	84.37 ± 10.44	81.5 ± 9.906	0.138
Neck circumference	39.28 ± 3.374^*^	37.58 ± 3.464^*^	33.48 ± 2.269	<0.001
Waist circumference	100.2 ± 8.886^*^^#^	94.53 ± 6.844^*^	76.89 ± 5.348	<0.001
Hip circumference	111.7 ± 9.668^*^^#^	106.3 ± 5.647^*^	93.3 ± 4.075	<0.001
WHR	0.8992 ± 0.060^*^	0.8895 ± 0.051^*^	0.8243 ± 0.048	<0.001
Insulin	22.93 ± 17.43^*^	19.52 ± 18.68	10.55 ± 13.24	0.015
FBG	5.339 ± 1.818	5.42 ± 1.185	4.743 ± 0.539	0.091
TC	4.644 ± 0.660	4.62 ± 0.796	4.248 ± 0.788	0.078
TG	1.521 ± 0.945^*^	1.427 ± 0.728^*^	0.930 ± 0.462	0.005
HDL-C	0.984 ± 0.126	1.014 ± 0.127	1.051 ± 0.1944	0.241
LDL-C	3.124 ± 0.635^*^	2.986 ± 0.672^*^	2.547 ± 0.7003	0.003
hs-CRP	4.25 ± 3.76^*^	4.777 ± 5.236^*^	1.563 ± 0.9554	0.003
UA	366.6 ± 93.29^*^	341.4 ± 88.83	286.6 ± 87.02	0.003
FFA	0.391 ± 0.151	0.389 ± 0.171	0.360 ± 0.150	0.703

Continuous, normally distributed variables among the three groups were analyzed by one-way analysis of variance. The Kruskal-Wallis H-test was applied for data of this type that was not normally distributed. The χ2 test compared categorical variables. Compared with the PH constitution group: *P<0.05; compared with the Non-PDC obesity group: ^#^P<0.05.

### Overview of the Gut Microbiome in Different Groups

**Figure 1:**
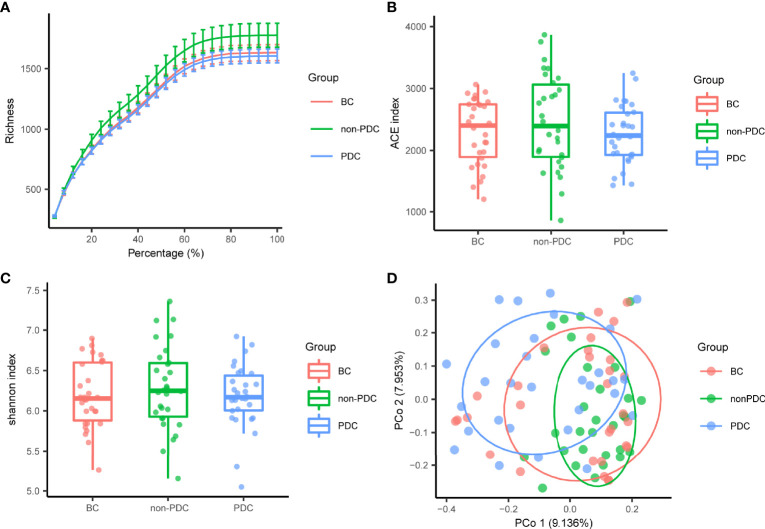
Gut microbiome diversity and structure analysis. **(A)** Rarefaction curve evaluating the relative bacterial richness to determine whether further sequencing would identify additional ASVs. BC, Balanced Constitution group; PDC, Obesity with phlegm-dampness constitution group; non-PDC: obesity without phlegm-dampness constitution group. **(B**, **C)** Species diversity differences among the PDC, non- PDC and BC groups were estimated by ACE index and Shannon index. **(D)** PCoA with Bray–Curtis distance showing that the overall microbial composition of three groups. BC (red dots); non-PDC group (green dots); PDC group (blue dots), where dots represent individual samples.

In our present microbiome investigation, the optimized reads ranging from 36,816 to 79,486 were obtained from all samples (**Additional File 2: Supplementary Table S1**). Following taxonomic assignment, 12061 ASVs) were obtained (**Additional File 2: Supplementary Table S2**). Rarefaction curves generated from the ASVs suggested that high sampling coverage (~99%) was achieved in all samples (**Figure 1A**). This indicated that the sequencing depth was sufficient for the investigation of the fecal microbiota. In terms of alpha diversity, we observed no significant differences in the ACE index (**Figure 1B**) or Shannon’s index (**Figure 1C**) between the three groups. To assess the overall structure of the gut microbiota, a score plot of PCoA based on the Bray-Curtis distances (**Figure 1D**) was constructed. The result revealed a separation of the gut microbiota structure of the PDC group and BC group (P = 0.033, PERMANOVAR by Adonis) or non-PDC (P<0.001, PERMANOVAR by Adonis). However, no significant difference in beta diversity was observed between the BC group and the non-PDC group. Our results showed that although the overall gut microbiota composition in the PDC group was different from both the BC and non-PDC groups, the overall structure of the gut microbiota in the non-PDC group was not significantly different from BC.

**Figure 2:**
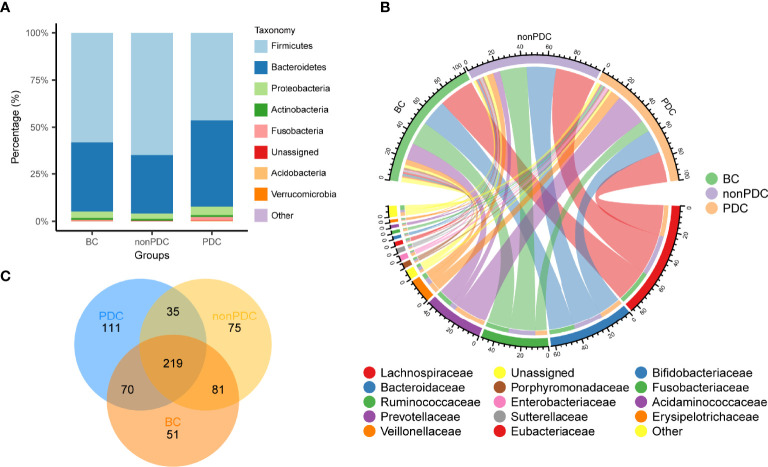
Overview of the gut microbiome in different groups. **(A)** Dominant phyla in each group. **(B)** Dominant families and their contribution to each group. **(C)** A Venn diagram demonstrating the existence of ASVs in each group.

The relative proportion of dominant taxa at the phylum level was assessed and 11 phyla were identified in each group (**Figure 2A**). *Firmicutes* was the most dominant phylum, with a relative abundance of 58.0% in the BC group, 64.8% in the non-PDC group and 46.4% in the PDC group. The second most dominant phylum was *Bacteroidetes* (control: 36.9%, non-PDC: 31.1%, PDC: 46.0%). Other observed phyla included *Proteobacteria, Actinobacteria*, *Fusobacteria, Verrucomicrobia*, and other phyla of extremely low abundance, including *Candidatus_Saccharibacteria, Acidobacteria, Tenericutes, and Lentisphaerae*. Several of the most abundant families and their contribution to each group are shown in **Figure 2B**. *Lachnospiraceae*, accounting for 28.6% of all samples, was the most predominant family. *Bacteroides* genus, accounted for 21.1% of the total, and was the predominant genus (**Figure S1A**). A Venn diagram was constructed to examine the existence of ASVs in each group (**Figure 2C**). Most ASVs (219 in all) were shared by all three groups. However, a total of 35 ASVs were specifically shared by two obesity groups. Additionally, a total of 81 ASVs were shared by only the BC group and the non-PDC group, and 70 ASVs were shared by only the BC and the PDC group. In total, 111 ASVs were uniquely present in the PDC group, 75 ASVs in the non-PDC group and 51 ASVs in the BC group.

### Alterations in the Composition of Fecal Microbiota Associated With PDC

**Figure 3:**
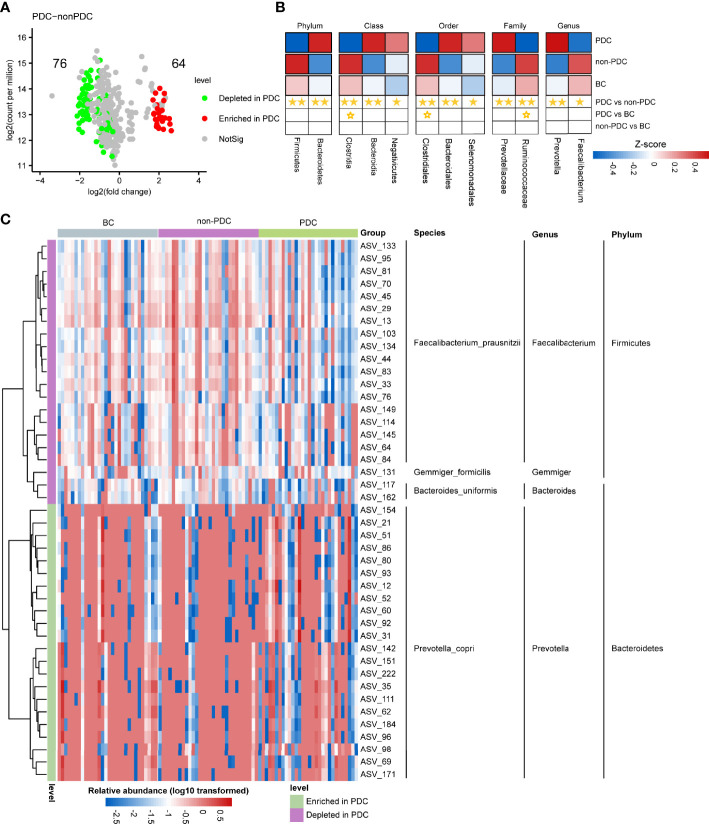
Alterations in the composition of fecal microbiota associated with PDC. **(A)** A volcano plot demonstrating differential ASVs between the PDC group and the non-PDC group. **(B)** A heatmap illustrating the relative abundance of PDC-associated taxa across the three groups (P value <0.05 by Wilcoxon rank sum test) and adjusted p<0.1 by FDR). The abundance profiles are transformed into Z scores by subtracting the average abundance and dividing the standard deviation of all samples. Z score is negative (shown in blue) when the row abundance is lower than the mean. Taxa at P value <0.01 are marked with two solid stars, P value <0.05 with one solid star, P value <0.1 with hollow star. **(C)** Heat map of the relative abundance of 43 ASVs with relative abundance greater than 0.1% that were significantly different between PDC group and non-PDC group (A threshold of P < 0.05 and FDR < 0.05 calculated by Wilcoxon rank sum test). ASVs are shown from lower abundance (in blue) to higher abundance (in red). All 43 ASVs were assigned to phyla, genera and species.

We utilized Wilcoxon rank-sum test to compare the differences in fecal microbiota at the ASV level between groups, a threshold of P < 0.05, FDR < 0.2 and the relative abundance greater than 0.1% across all samples were selected. We mainly focused on the significantly different taxa between the PDC group and the non-PDC group, since we considered these taxa to be associated with obesity of different types of constitutions. As shown in the volcano plot (**Figure 3A**), a total of 140 ASVs exhibited significantly different abundances in the two obesity groups, including 64 ASVs enriched in the PDC group and 76 ASVs depleted in the PDC group. The heat map shows the abundance of differential ASVs with relative abundance greater than 0.1% across all samples and the adjusted p < 0.05 (**Figure 3C**). The ASVs enriched in PDC all belonged to *Bacteroidetes* phylum, *Prevotella* genus, *Prevotella_copri* species. Except for ASV117, ASV162 belonging to the *Bacteroides* genus and ASV137 belonging to the *Gemmiger* genus, most of the ASVs depleted in the PDC belonged to the *Firmicutes* phylum, *Faecalibacterium* genus, and *Faecalibacterium_prausnitzii* species. Compared with the BC group, the abundance of 67 ASVs in the PDC group significantly altered, of which 25 were increased and 42 were decreased (**Figure S1B**). However, compared with the people with BC, obese people with non-PDC showed no significant alterations in the abundance (**Figure S1C**, **Additional File 2: Supplementary Table S3**). To investigate the specific changes of microbiota in samples from the PDC group, we assessed the relative abundance of bacterial species across three groups (**Figure 3B**). At the phylum level, *Firmicutes* was significantly more decreased in the PDC group than in the non-PDC group (P<0.001, Wilcoxon rank sum test adjusted by FDR). Compared to the non-PDC group, the PDC group was characterized by higher *Bacteroidetes* levels (P<0.001) and a significantly lower *Firmicutes/Bacteroidetes* ratio (P<0.001). In agreement with the findings at phylum level, we found differential abundance of dominant classes, orders, families, genera in the fecal microbiota between PDC and non-PDC samples. At the family level, we noted that *Ruminococcaceae* (PDC vs non-PDC: P=0.002, PDC vs BC: P= 0.071), typically producing short chain fatty acids (Cheng et al., 2021), depleted in the PDC group. Instead, *Prevotellaceae* was significantly enriched in the PDC group (PDC vs non-PDC: P= 0.002). Butyrate-producing *Faecalibacterium* bacteria depleted in the PDC group (PDC vs non-PDC: P= 0.042), however, *Prevotella* enriched in the PDC group (PDC vs non-PDC: P= 0.004). In addition, we identified 19 bacterial species that showed nominal alterations in the PDC group (P value <0.05 by Wilcoxon rank sum test, and adjusted p >0.05 by FDR), of interest, *Prevotella_copri* and *Faecalibacterium_prausnitzii* undergo more significant changes in PDC (adjusted p <0.1 by FDR), which both them considered influence the metabolic state of the host in previous studies, but limited by 16S amplicon sequencing, which can only partially explain the disturbed gut microbiota in PDC.

### Relationship Between the Gut Microbiota and Obesity Phenotypes Associated With Different Constitutions

**Figure 4:**
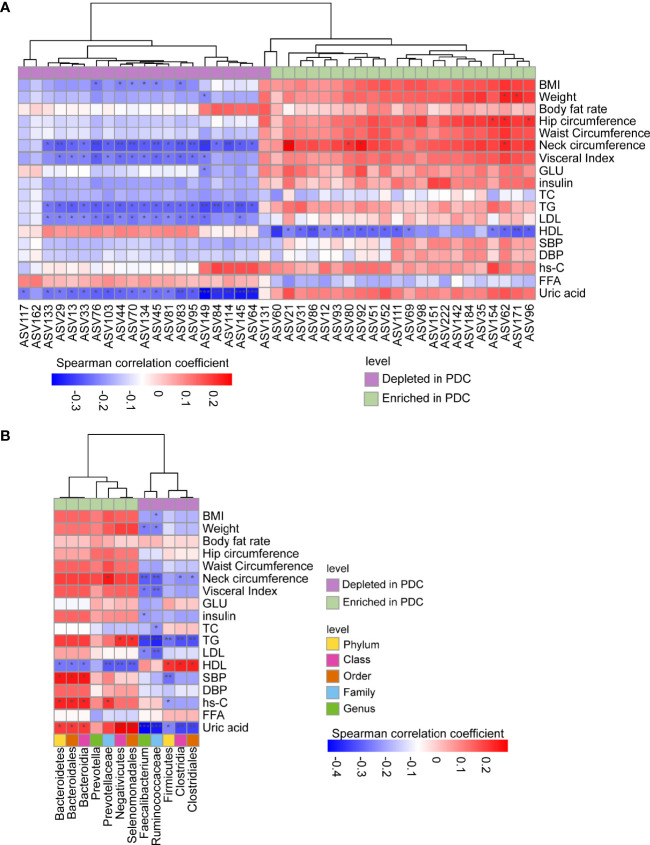
Spearman correlations between PDC-associated gut flora and clinical phenotypes. ASVs or taxa enriched in the PDC group were labeled with light green, while depleted ones with purple. **(A)** Correlations between PDC-associated ASVs and phenotypes. **(B)** Correlations between discrepant taxa and phenotypes. Note: *, **, *** represent p<0.05, p<0.01, p<0.001, respectively.

Furthermore, we correlated the PDC-associated ASVs and bacterial taxa to clinical phenotypes using the Spearman correlation method (**Figures 4A, B**). A total of 35 ASVs were found to be significantly correlated with clinical phenotypes (**Figure 4A**). Notably, all ASVs belonging to *Faecalibacterium*, depleted in PDC, were significantly negatively correlated with neck circumference, TG, uric acid, and most were negatively correlated with LDL, visceral index, and BMI. In contrast, most ASVs enriched in PDC group, annotated as *Prevotella*, were negatively correlated with HDL, and a small number of ASVs were significantly negatively correlated with obesity indicators such as weight, hip circumference, and neck circumference.

A total of 49 correlations were found between different levels of PDC-associated bacteria and clinical phenotypes (**Figure 4B**). Of these, only *Ruminococcaceae* were associated with BMI, which is a clinical diagnostic indicator of obesity. Correlations between PDC-related taxa and TG, HDL, SBP, hs-C, and uric acid, all of which are indicators associated with metabolic diseases, deserve more attention. For example, species enriched in PDC were significantly positively correlated with TG, SBP, hs-C and uric acid, however, these bacteria did not correlate significantly with obesity-related indicators such as BMI and waist circumference. Also, species depleted in PDC were significantly negatively correlated with TG, where Firmicutes were only correlated with HDL, uric acid and other metabolism-related phenotypes, but not with obesity-related phenotypes. In addition, uric acid and HDL are most closely related to altered intestinal bacteria and deserve focused attention. These results suggest that changes in PDC-associated bacterial taxa carry a higher metabolic risk compared to more severe obesity.

### Prediction of Metagenomic Functional Changes Associated With Obesity With Different Constitutions

The metagenomic pathways were predicted using the PICRUSt tool based on the KEGG database. All total of 70 pathways were found to differ in abundance between the PDC group and the non-PDC group (P < 0.05, Welch’s t-test, FDR < 0.05, **Figure S2**; **Table S4**) (40 pathways enriched in the PDC group and 30 pathways depleted in the PDC group).

Furthermore, we correlated the PDC-associated pathways and PDC-associated ASVs with relative abundance greater than 0.1% using the Spearman correlation method (**Figure S3**; **Additional file 2: Supplementary Table S5**). ASVs enriched in PDCs seem to have a greater effect on metabolic pathways, and most especially have a strong positive correlation with arachidonic acid metabolism and mineral absorption. In addition, we found very interesting correlations between PDC-related differential metabolic pathways and differential bacterial species. There was a consistency in the trends of KOs and PDC-related bacterial species changes. We hypothesize that it is the differences in these particular gut microbes that lead to the significant differences in predicted metagenomic function. Among them, protein digestion and absorption were strongly associated with PDC-associated taxon, especially with *Bacteroidia* significantly and positively (r=0.912, P<0.001). *Bacteroidia* taxon were significantly and positively correlated with cellular antigens. *Prevotella* was positively correlated with mineral absorption (**Figure S4**; **Additional File 2: Supplementary Table S6**). Minerals, carbohydrates, and proteins are food nutrients, and they are the main stimulators of glucagon-like peptide-1 (GLP-1), which over production can disrupt glucose homeostasis (Qin et al., 2021). The lipopolysaccharide biosynthesis and lipopolysaccharide biosynthesis proteins pathways, which were significantly enriched in PDCs, are recognized pro-inflammatory factors that disrupt the intestinal barrier and cause metabolic disturbances (Fabbiano et al., 2018); however, they are negatively correlated with bacteria enriched in PDCs and positively correlated with bacteria reduced in PDCs. It is worth mentioning that arachidonic acid metabolism; glycine, serine and threonine metabolism, and other pathways are enriched in PDC; meanwhile, arginine and proline metabolism; flavone and flavonol biosynthesis; glycolysis/gluconeogenesis; primary bile acid biosynthesis; metabolic pathways such as secondary bile acid biosynthesis; insulin signaling pathway are depleted in PDC (**Figure S2**).

### Identification of Obesity With PDC Basing on Gut Microbiome

**Figure 5:**
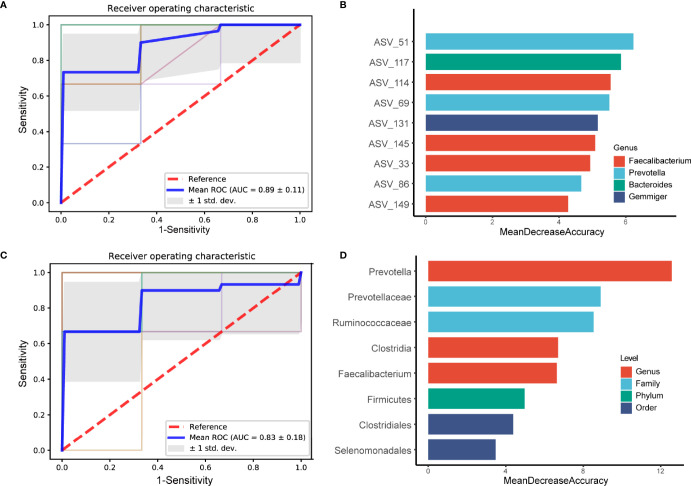
Diagnostic outcomes are shown *via* receiver operating characteristic (ROC) curves for PDC and BC. A, C. Random Forest models are constructed using explanatory variables of ASVs **(A)** and taxon **(C)**. **(B, D)** The detailed explanatory variables based on the random forest model in each comparison. The lengths of the bars in the histogram represent the mean decrease accuracy, which indicates the importance of the ASVs or taxon for classification.

To exploit the potential of gut microbiome in obese people with PDC identification, random forest classifier using explanatory variables of ASVs and species abundances were performed. Tenfold cross-validation was repeated and the receiver operating characteristic (ROC) curves for classifying people with PDC from all obese individuals. We could detect PDC individuals accurately based on the gut ASVs, as indicated by the area under the receiver operating curve (AUC) of up to 0.89 ± 0.11 (**Figure 5A**). Thus, we conducted a testing set consisted of 20 randomly chosen obese subjects based on ASVs. In this assessment analysis, PDC possesses remarkable features in gut microbiome as compared to the non-PDC (AUC=0.80 ± 0.40) (**Figure S5A**). Among the strongest discriminatory features, ASV51 from *Prevotella* genus had the greatest impact, followed by characteristics such as ASV117(*Bacteroides*), ASV114(*Faecalibacterium*), ASV69(*Prevotella*) (**Figure 5B**). We also investigated the utility of the classifier based on microbial taxa. Consistently, the AUC for identifying PDC from the obese people was 0.83 ± 0.18 (**Figure 5C**). *Prevotella*, *Prevotellaceae, Ruminococcaceae, Clostridia, Faecalibacterium* were the strongest discriminatory features (**Figure 5D**). Similarly, we conducted a test group of 20 randomly selected obese subjects according to microbial taxa, the AUC was 0.70 ± 0.46 (**Figure S5B**). Overall, the PD-associated microbial features captured by the classifier offered further evidence of the dysbiosis gut microbiome and highlighted its great potential for distinction of obesity with different constitutions.

## Discussion

Obesity is a complex condition commonly associated with metabolic abnormalities. To date, there are various methods to measure obesity subtypes, but they cannot accurately predict the metabolic risk associated with obesity. Therefore, to achieve precise control of obesity-associated metabolic abnormalities, new obesity typing methods need to be explored. Phlegm-damp constitution, a concept specific to TCM, is considered an obese subtype with high metabolic risk, and the risk of metabolic abnormalities associated with obesity can be reduced by specific interventions for phlegm-damp constitution. In the present study, we aimed to further understand the role played by PDC in obesity and its complications by exploring PDC-related microbiome changes in obese patients with phlegm-dampness.

Overall, although we observed no significant differences in the abundance of gut bacteria between groups according to the alpha diversity analysis, the beta diversity analysis showed that significant differences in microbial composition in both the PDC group and the non-PDC group/BC group. However, there was no significant difference in beta diversity between the non-PDC group and BC, suggesting variation in PDC-associated gut microbiota compared to obese non-PDC and healthy individuals, but the non-PDC obese population are similar to healthy individuals. Furthermore, we focused on specific taxa changes associated with PDC. In our study, the *Firmicutes/Bacteroidetes* ratio was decreased in the PDC group. This ratio was elevated in obesity (Chang et al., 2015), hypertension (Li et al., 2017), and autism (Dan et al., 2020), and decreased in a Hypoxia Induced Factor 1α (HIF-1α) induced alcoholic liver disease mouse model (Shao et al., 2018). *Bacteroidetes*, which are enriched in PDC, are the most abundant bacterial phylum in the human intestine. It is involved in the fermentation of polysaccharide, the utilization of nitrogenous substances, the production of propionates, and is often portrayed as a beneficial bacterium in previous studies (Hayden et al., 2020; Sequeira et al., 2020). However, it is also involved in the release of toxic substances during proteolysis, which promotes inflammation (Jia et al., 2021). In our study, the *Bacteroidetes* enriched in PDC, which was positively correlated with serum C-reactive protein and systolic blood pressure and negatively correlated with HDL, plays a negative factor for obesity and its complications. *Firmicutes* depleted in PDC plays an important role in polysaccharide breakdown and production of short-chain fatty (SFCA) acids, many different species of bacteria within several families belonging to the phylum *Firmicutes* have been identified as SCFAs producers, mainly acetate, propionate, and butyrate, which are anti-inflammatory and improve metabolism effects through reduction of pro-inflammatory factors in the blood, such as LPS (Fernández et al., 2016). Interestingly, the results of the correlation analysis between *Firmicutes* and clinical phenotypes were opposite to those of *Bacteroidetes*, with the former enriched with PDC associated with lower SBP, triglycerides, and uric acid, and higher HDL-C. In other words, *Firmicutes* played a probiotic role in this cohort.

More interesting were the dysbiosis patterns. We found that many of the microbial taxon with altered abundance in the PDC group were associated with the production of SCFAs, plasma bile acid, and anti-inflammatory protection according to several independent studies. *Ruminococcaceae* depleted in PDC could ferment fiber and other plant components of the diet such as inulin and cellulose, producing short-chain fatty acids (SCFA) which can both be utilized by the host for energy and display anti-inflammatory properties in the gut (Astbury et al., 2020). Cold exposure reduced high-fat diet-induced obesity in mice Their gut microbiotawere characterized by increased levels of *Ruminococcaceae*, which may be associated with increased iBAT thermogenesis and a plasma bile acid profile (Ziętak et al., 2016). In obese individuals, *Prevotellaceae* enrichment was elevated, producing circulating succinate, which is a potential microbiota-derived metabolite related to CVD risk (Serena et al., 2018). Notably, and this is consistent with our study, *Prevotellaceae* were enriched in PDC and were positively correlated with neck circumference and negatively correlated with HDL-C. At the genus level, *Faecalibacterium*, which is involved in producing intestinal epithelial nutrition, producing short-chain fatty acid, achieving anti-inflammatory effects, and strengthening intestinal barrier functions (Hiippala et al., 2018; Chen et al., 2020), were depleted in obese patients with PDC. Further exploration revealed the depleted *Faecalibacterium_prausnitzii* in the PDC group, which was found to exert anti-inflammatory activity both *in vitro* and *in vivo (* Llopis et al., 2016; Quévrain et al., 2016). In our study, *Prevotella* enriched in PDC is one dominant genus of *Bacteroidetes*, however, it had contradictory results on host metabolic effects in previous studies. Elevated abundance of *Prevotella* is usually associated with dietary fiber-rich dietary interventions, and enrichment of *Prevotella* can promote weight loss, lower cholesterol levels, and improve glucose metabolism (Kovatcheva-Datchary et al., 2015; Ortega-Santos and Whisner, 2019). By contrast, another study found that *Prevotella copri*, was identified as the primary species driving the association between biosynthesis of branched-chain amino acids (BCAAs) and insulin resistance, inducing insulin resistance, exacerbating glucose intolerance, and increasing circulating BCAAs levels (Pedersen et al., 2016), and the abundance of *Prevotella copri* potentially attenuate the protective effect of the Mediterranean diet on reduction of insulin resistance and cardiometabolic disease risk (Meslier et al., 2020; Wang et al., 2021).Based on the above analysis, we hypothesize that bacteria involved in the synthesis of SFCAs and bile acids are in reduced abundance in PDCs, thus promoting inflammation and increasing the risk of metabolic disorders. However, the above ideas need to be validated by metagenomic sequencing studies with larger sample sizes.

This is emphasized by the predicted gene function based on 16srDNA amplicon sequencing data. The PICRUSt analysis demonstrated that several microbial functions were significantly over- or underrepresented between groups, due to important differences in bacteria composition. Compared with non-PDC people, gut microbiota in PDC individuals had a depleted abundance of genes involved in metabolic pathways such as arginine and proline metabolism, flavone and flavonol biosynthesis, glycolysis/gluconeogenesis, and primary bile acid biosynthesis. Conversely, there was an increase in genes related to arachidonic acid metabolism, glycine, serine and threonine metabolism, the lipopolysaccharide biosynthesis pathway, related to inflammation and immune response. The relative abundance of genes associated with a given pathway may indicate an increased metabolic capacity of the gut microbiota with regard to this pathway. Disturbances in the metabolism of arginine and proline associated with urinary phthalate exposure may contribute to the development of overweight and obesity in school-aged children (Xia et al., 2018). Flavonoids, plant-derived polyphenolic compounds, have been linked with health benefits. For the gastrointestinal tract, it can modulate the secretion of gut hormones, immune system, shape microbiota composition and function, and maintain the intestinal barrier integrity. Importantly, flavonoid actions at the GI tract can have an impact systemically, e.g., on glucose homeostasis, lipid and energy metabolism, or cardiovascular risk factors (Oteiza et al., 2018). Arachidonic acid metabolism has a higher proportion in the PDC group, which is an unsaturated fatty acid, which entails risk for developing intestinal inflammation, obesity, type 1 diabetes, and alcoholic fatty liver (Leiva-Gea et al., 2018; Miyamoto et al., 2019; Mayr et al., 2020; Sun et al., 2020). LPS, a microbial cell wall fraction, is capable of disrupting the intestinal barrier and triggering systemic low-grade inflammation associated with metabolic syndrome, visceral fat mass, and type 1 diabetic nephropathy (Leiva-Gea et al., 2018).

In addition, it is important to mention that waist circumference, hip circumference, and BMI were higher in PDC individuals compared to non-PDC obese individuals, which is similar to previous studies that abdominal and pear-shaped obese individuals who hoard more white fat are at higher risk for metabolic disease (Quan et al., 2020). However, PDC is not the same as abdominal obesity. The constitution is a comprehensive classification method that includes three dimensions of body form, physiological function, and psychological state, and excessive abdominal fat is only one of the characteristics of PDC (Li et al., 2021). In our study, *Ruminococcaceae* and several ASVs annotated as *Faecalibacterium*, depleted in PDC group, were negatively correlated with BMI, the other PDC-associated ASVs and bacterial species were not correlated with BMI and waist circumference, the two keys to the diagnosis of obesity. Most of the bacterial species associated with PDC were more strongly correlated with indicators of metabolic disease diagnosis such as TG, HDL, hs-c, uric acid. Notably, ASVs enriched in PDC group had a stronger correlation with HDL, whereas ASVs reduced in PDC had a stronger correlation with TG, LDL, and uric acid. *Bacteroidetes* and *Firmicutes* were correlated with HDL, TG, hs-c and Uric acid, and were not associated with obesity-related indicators. These results suggest that changes in PDC-associated bacterial taxa carry a higher metabolic risk rather than more severe obesity.

In general, evidence that obese patients with PDC have a higher risk of inflammatory and metabolic diseases can be observed from the perspective of intestinal microbiota. Our study suggests that gut microbial characteristics can also be used as biomarkers to microscopically differentiate between obese individuals with PDC or non-PDC.

Our study has some limitations. Although our study showed that the gut microbiota of obese patients with PDC may have a higher risk of metabolic disorders, we only assessed microbial characteristics at the same time and did not monitor dynamic changes, which need to be further explored in future longitudinal studies. Second, the effects of many confounding factors were not recorded, such as diet and lifestyle, which may lead to bias in the correlation analysis. In addition, data interpretation may be limited by the relatively small sample size and amplicon sequencing-based analysis of the gut microbiome, and conclusions need to be validated by larger metagenomic studies.

## Conclusion

Taken together, our study suggests that the composition of the gut microbiota differs between obese individuals with PDC and non-PDC subjects. Alterations in the gut microbiota of the obese population with the two PDCs were associated to a greater extent with more metabolic disturbances rather than more severe obesity. Such alterations in the gut microbiota may lead to systemic metabolic disturbances by affecting bacterial inflammatory and immune responses, polysaccharide, carbohydrate, and amino acid metabolism, etc. In addition, specific bacterial signatures have shown potential diagnostic value in differentiating obese individuals with different constitutions. These findings may provide a microbiome component to the TCM view that obese individuals with PDC are at high risk for metabolic disease, and also provide a new perspective on obesity typing to aid in more precise prevention and treatment of obesity.

## Data Availability Statement

The datasets presented in this study can be found in online repositories. The names of the repository/repositories and accession number(s) can be found below: https://www.ncbi.nlm.nih.gov/, accession ID: PRJNA810767.

## Ethics Statement

The studies involving human participants were reviewed and approved by the Ethics Committee of Beijing University of Chinese Medicine. The patients/participants provided their written informed consent to participate in this study.

## Author Contributions

JS, TL, and LZ: study design, data collection, analysis, and writing. QW: study design, give direction to the paper. XL: manuscript writing and revising. YNL and SZ: data collection and analysis. XW, YSL and LL: study design, give direction to the paper. All authors contributed to the articles and approved the submitted version.

## Funding

This work was supported by the State Key Program of National Natural Science of China (81730112 to QW), Beijing Nova Program (Z201100000820027 to LL), and Innovation Team Project of Beijing University of Chinese Medicine (2019-JYB-TD010 to LL).

## Conflict of Interest

Author YNL was employed by School of Traditional Chinese Medicine, Beijing University of Chinese Medicine. YNL was employed by People’s Medical Publishing House Co., Ltd.

The remaining authors declare that the research was conducted in the absence of any commercial or financial relationships that could be construed as a potential conflict of interest.

## Publisher’s Note

All claims expressed in this article are solely those of the authors and do not necessarily represent those of their affiliated organizations, or those of the publisher, the editors and the reviewers. Any product that may be evaluated in this article, or claim that may be made by its manufacturer, is not guaranteed or endorsed by the publisher.

## Abbreviations

FBG, fasting blood glucose; TC, total cholesterol; TG, triglyceride; HDL-C, high-density lipoprotein cholesterol; LDL-C, low-density lipoprotein cholesterol; FFA, free fatty acids; hs-CRP, hypersensitive-c-reactive-protein; UA, uric acid; SBP, systolic blood pressure; DBP, diastolic blood pressure; BMI, body mass index.
