# Supplementary material for: Obese Individuals With and Without Phlegm-Dampness Constitution Show Different Gut Microbial Composition Associated With Risk of Metabolic Disorders
Source: Front Cell Infect Microbiol. 2022 Jun 1;12:859708. doi: 10.3389/fcimb.2022.859708 (PMC9199894; doi:10.3389/fcimb.2022.859708)
Supplement: Supplementary file 1 [file DataSheet_1.docx]

**Additional file 3**

**Supplementary Methods**

**Obese individuals with and without Phlegm-dampness Constitution Show different Gut Microbial Composition associated with risk of metabolic disorders**

**Wang Qi’s Body Constitution Classification Questionnaire**

This questionnaire aims to survey your constitution and to subsequently provide a reference for future health management and clinical diagnosis. Please read every question carefully and choose the most suitable response based on your actual situation or experience in the past year. If you are unsure of the answer to a specific question, choose the answer that is most similar to your actual situation. Make sure that you answer all the questions based on your situation **in the past year**, (excluding the effect of drugs) and give only one answer to each question.

Explain:【No】Never happened in the past year.【Slightly】Occasionally happened in the past year.【Sometimes】Sometimes it happened, but no regular pattern. 【Often】It happened most of the time in the past year. 【All the time】It happened all the time in the past year.

Experience/condition in the past year No Slightly Sometimes Often All the time

| **Experience/condition in the past year** | **No** | **Slightly** | **Sometimes** | **Often** | **All the time** |
| --- | --- | --- | --- | --- | --- |
| (1) Were you energetic? | 1 | 2 | 3 | 4 | 5 |
| (2) Did you get tired easily? | 1 | 2 | 3 | 4 | 5 |
| (3) Did you experience shortness of breath? | 1 | 2 | 3 | 4 | 5 |
| (4) Did you get palpitations? | 1 | 2 | 3 | 4 | 5 |
| (5) Did you get dizzy easily or become dizzy when standing up? | 1 | 2 | 3 | 4 | 5 |
| (6) Did you prefer quietness and not like to talk? | 1 | 2 | 3 | 4 | 5 |
| (7) Was your voice weak when talking? | 1 | 2 | 3 | 4 | 5 |
| (8) Did you feel in low spirits and depressed? | 1 | 2 | 3 | 4 | 5 |
| (9) Did you easily feel anxious and worried? | 1 | 2 | 3 | 4 | 5 |
| (10) Did you feel overly sensitive, vulnerable or emotionally upset? | 1 | 2 | 3 | 4 | 5 |
| (11) Were you easily scared or frightened? | 1 | 2 | 3 | 4 | 5 |
| (12) Did you experience distention in the underarm or breast? | 1 | 2 | 3 | 4 | 5 |
| (13) Did you feel chest or abdominal stuffiness? | 1 | 2 | 3 | 4 | 5 |
| (14) Did you sigh without reason? | 1 | 2 | 3 | 4 | 5 |
| (15) Did your body feel heavy or lethargic? | 1 | 2 | 3 | 4 | 5 |
| (16) Did the palms of your hands or soles of your feet feel hot? | 1 | 2 | 3 | 4 | 5 |
| (17) Did your hands or feet feel cold or clammy? | 1 | 2 | 3 | 4 | 5 |
| (18) Did you feel cold easily in your abdomen, back, lower back or knees? | 1 | 2 | 3 | 4 | 5 |
| (19) Were you sensitive to cold and tended to wear more clothes than others? | 1 | 2 | 3 | 4 | 5 |
| (20) Did your body and face feel hot? | 1 | 2 | 3 | 4 | 5 |
| (21) Did you feel more vulnerable to the cold than others (winter coldness, air conditioners, fans, etc.)? | 1 | 2 | 3 | 4 | 5 |
| (22) Did you catch colds more easily than others? | 1 | 2 | 3 | 4 | 5 |
| (23) Did you sneeze even when you did not have a cold? | 1 | 2 | 3 | 4 | 5 |
| (24) Did you have a runny or stuffy nose even when you did not have a cold? | 1 | 2 | 3 | 4 | 5 |
| (25) Did you cough due to seasonal changes, temperature changes, or unpleasant odors? | 1 | 2 | 3 | 4 | 5 |
| (26) Did you sweat easily when your physical activity increased slightly? | 1 | 2 | 3 | 4 | 5 |
| (27) Did you forget things easily? | 1 | 2 | 3 | 4 | 5 |
| (28) Did you have an excessively oily forehead and/or T-zone? | 1 | 2 | 3 | 4 | 5 |
| (29) Were your lips redder than in the past? | 1 | 2 | 3 | 4 | 5 |
| (30) Did you have allergies? (e.g. medicine, food, odors, pollen, pet dander, or during seasonal or weather change etc.) Experience/condition in the past year No Slightly Sometimes Often All the time | 1 | 2 | 3 | 4 | 5 |
| (31) Did you get hives/urticaria easily? | 1 | 2 | 3 | 4 | 5 |
| (32) Did your skin have purpura (purple spots, ecchymosis) due to allergies? | 1 | 2 | 3 | 4 | 5 |
| (33) Did black or purple bruises appear on your skin for no reason? | 1 | 2 | 3 | 4 | 5 |
| (34) Did your skin turn red and show traces when you scratched it? | 1 | 2 | 3 | 4 | 5 |
| (35) Did your skin or lips feel dry? | 1 | 2 | 3 | 4 | 5 |
| (36) Did you have visible capillary (thread) veins on your cheeks? | 1 | 2 | 3 | 4 | 5 |
| (37) Did you feel pain somewhere in your body? | 1 | 2 | 3 | 4 | 5 |
| (38) Did you experience hot flashes? | 1 | 2 | 3 | 4 | 5 |
| (39) Did your nose or your face feel greasy, oily, or shiny? | 1 | 2 | 3 | 4 | 5 |
| (40) Did you have a dark face or get brown spots easily? | 1 | 2 | 3 | 4 | 5 |
| (41) Did you get acne or sores easily? | 1 | 2 | 3 | 4 | 5 |
| (42) Did you have upper eyelid swelling? | 1 | 2 | 3 | 4 | 5 |
| (43) Did you get dark circles under the eyes easily? | 1 | 2 | 3 | 4 | 5 |
| (44) Did your eyes feel dry and you used eye drops? | 1 | 2 | 3 | 4 | 5 |
| (45) Were your lips darker, more blue or purple than usual? | 1 | 2 | 3 | 4 | 5 |
| (46) Did you often feel parched and need to drink water? | 1 | 2 | 3 | 4 | 5 |
| (47) Did your throat feel strange (i.e., as if something was stuck or there was a lump in your throat)? | 1 | 2 | 3 | 4 | 5 |
| (48) Did you have a bitter or strange taste in your mouth? | 1 | 2 | 3 | 4 | 5 |
| (49) Did your mouth feel sticky? | 1 | 2 | 3 | 4 | 5 |
| (50) Was your abdomen flabby? | 1 | 2 | 3 | 4 | 5 |
| (51) Did you have an abundance of phlegm, especially in your throat? | 1 | 2 | 3 | 4 | 5 |
| (52) Did you feel uncomfortable when you drank or ate something cold, or did you avoid to drinking or eating cold items? | 1 | 2 | 3 | 4 | 5 |
| (53) Could you adapt yourself to external natural or social environment changes? | 1 | 2 | 3 | 4 | 5 |
| (54) Did you easily experience insomnia? | 1 | 2 | 3 | 4 | 5 |
| (55) Did you easily contract diarrhea when you were exposed to cold or ate (or drank) something cold? | 1 | 2 | 3 | 4 | 5 |
| (56) Did you pass sticky stools and/or feel that your bowel movement was incomplete? | 1 | 2 | 3 | 4 | 5 |
| (57) Did you get constipated easily or have dry stools? | 1 | 2 | 3 | 4 | 5 |
| (58) Did your tongue have a thick coating? | 1 | 2 | 3 | 4 | 5 |
| (59) Did your urethral canal feel hot when you urinated, or did your urine have a dark color? | 1 | 2 | 3 | 4 | 5 |
| (60) Was your vaginal discharge yellowish (only for female interviewees)? | 1 | 2 | 3 | 4 | 5 |
| (60) Was your scrotum always wet (only for male interviewees)? | 1 | 2 | 3 | 4 | 5 |

**Metadata collection**

The collected metadata covered participants’ anthropometric features and information related to health status, disease history, medication, etc. Participants' body mass index (BMI) was calculated as weight (kg) divided by squared height (m2). Waist circumference was measured by circling the abdomen in a horizontal direction 5 cm above the umbilicus. Hipline was measured at the most prominent part of the buttocks. The neck circumference is measured horizontally around the upper edge of the Adam's apple. The body fat percentage (%) and visceral fat index were estimated by a fat meter (HBF-371, OMRON). For the participants, peripheral fasting blood was drawn in the morning. Peripheral blood samples were centrifuged at 3000 rpm for 5 min after standing at room temperature for at least 30 min, and the supernatant was purified. Then, the laboratory data were measured immediately. The detected indicators include 1) Indexes related to glucolipid metabolism, including insulin, FBG, TC, TG, HDL-C, LDL-C; 2）Inflammation-related biomarkers, including CRP, UA, FFA.
