# Supplementary figures and images for: Obese Individuals With and Without Phlegm-Dampness Constitution Show Different Gut Microbial Composition Associated With Risk of Metabolic Disorders
Source: Front Cell Infect Microbiol. 2022 Jun 1;12:859708. doi: 10.3389/fcimb.2022.859708 (PMC9199894; doi:10.3389/fcimb.2022.859708)

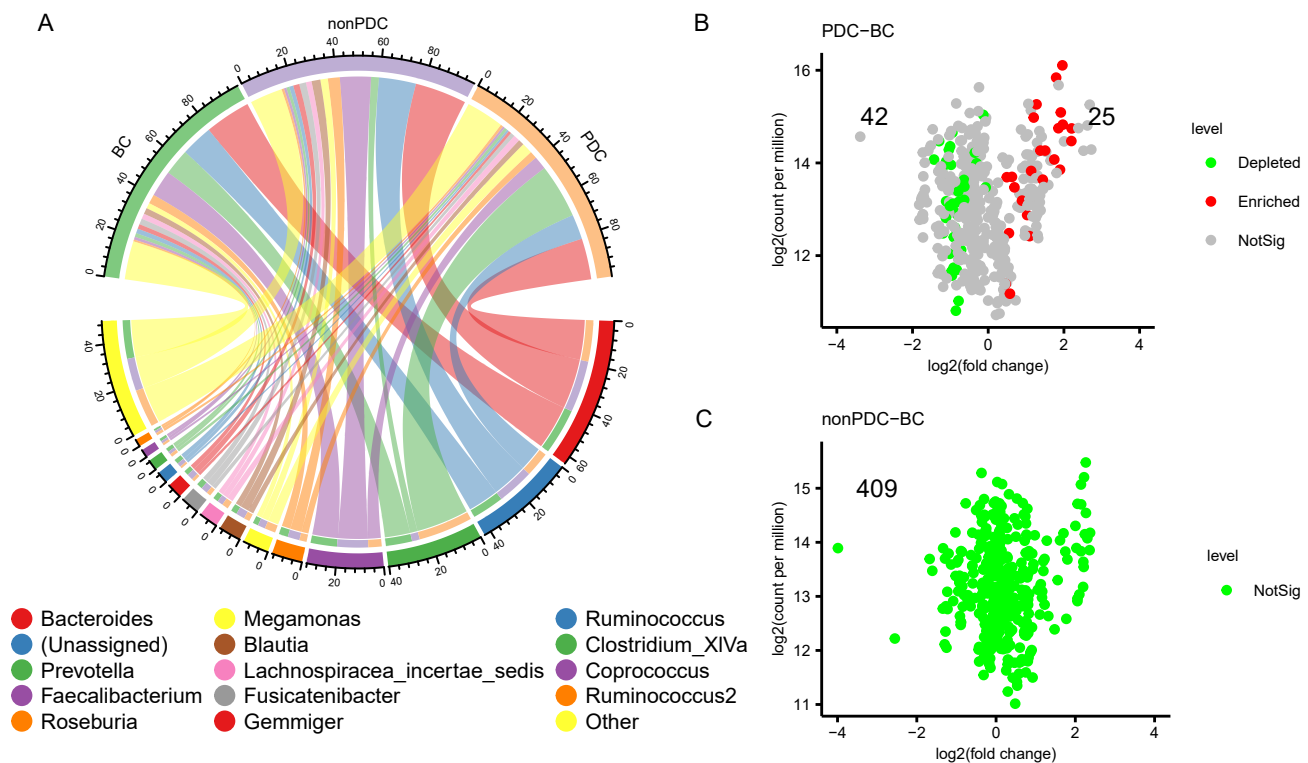

Supplementary Figure 1

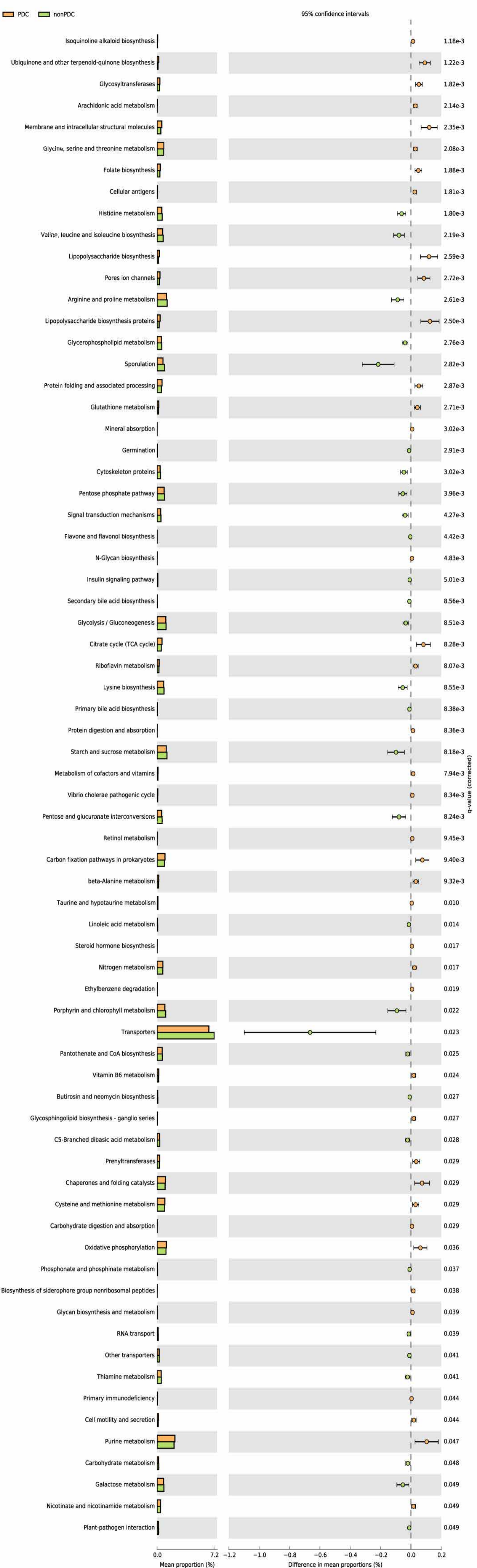

Supplementary Figure2

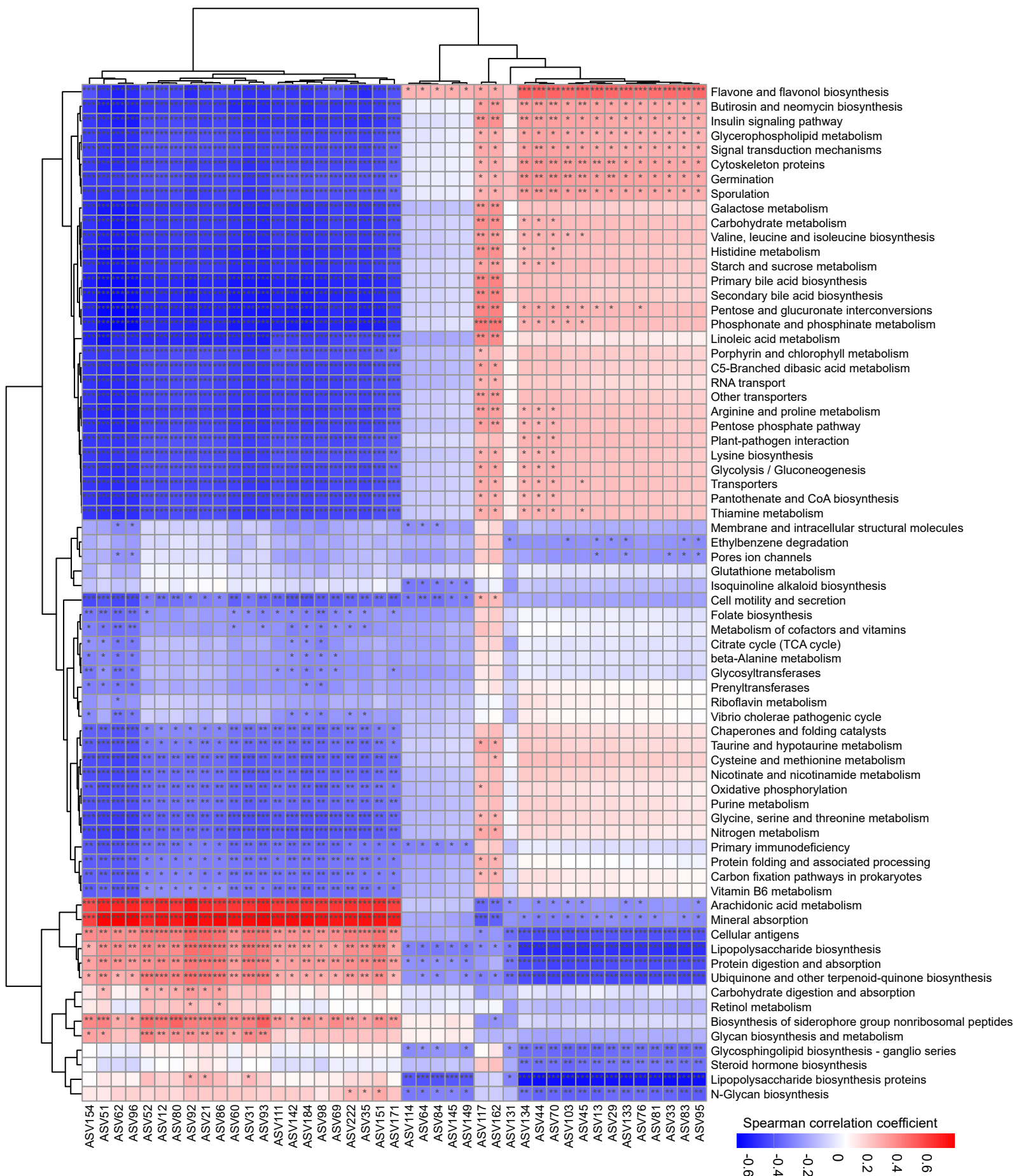

Supplementary Figure 3

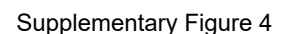

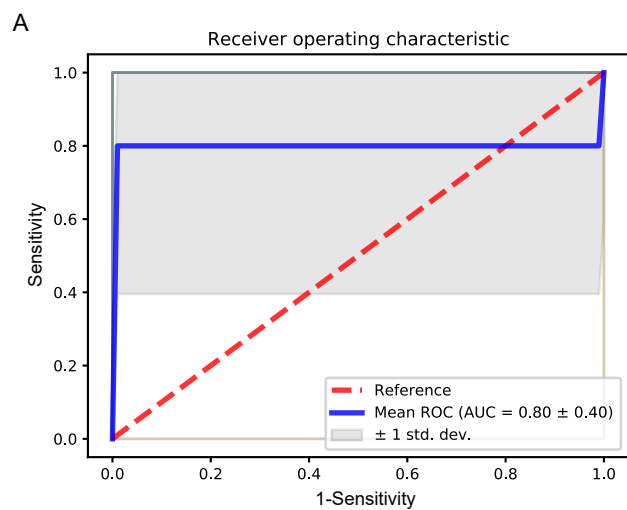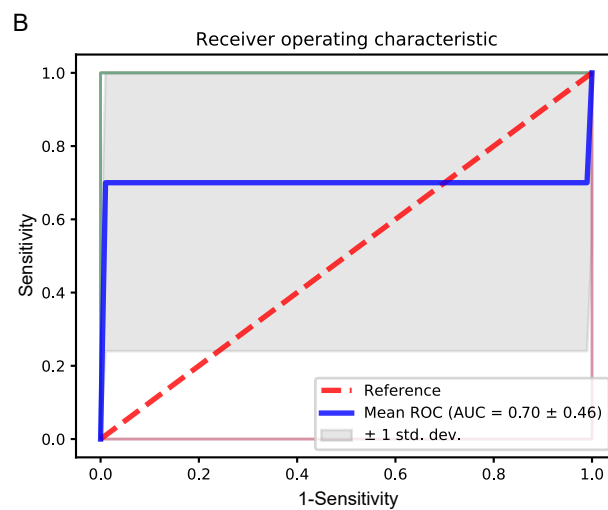

Supplementary Figure 5

Supplement: Supplementary file 2 [file DataSheet_2.pdf]
